# 1-Iodohexadecane Alleviates 2,4-Dinitrochlorobenzene-Induced Atopic Dermatitis in Mice: Possible Involvements of the Skin Barrier and Mast Cell SNARE Proteins

**DOI:** 10.3390/molecules27051560

**Published:** 2022-02-25

**Authors:** Do Yoon Kim, Kyung-Jong Won, Dae Il Hwang, Nan Young Kim, Bokyung Kim, Hwan Myung Lee

**Affiliations:** 1 Division of Cosmetic and Biotechnology, College of Life and Health Sciences, Hoseo University, Asan 31499, Korea; doyoon@hoseo.edu (D.Y.K.); bosshdi@hanmail.net (D.I.H.); rlasksdud01@naver.com (N.Y.K.); 2 Department of Physiology and Medical Science, School of Medicine, Konkuk University, Seoul 05029, Korea; kjwon@kku.ac.kr (K.-J.W.); bkkim2@kku.ac.kr (B.K.)

**Keywords:** 1-iodohexadecane, atopic dermatitis, SNARE protein, mast cells, degranulation, skin barrier protein

## Abstract

Atopic dermatitis (AD) is a chronic inflammatory dermal disease with symptoms that include inflammation, itching, and dry skin. 1-Iodohexadecane is known as a component of *Chrysanthemum boreale* essential oil that has an inhibitory effect on AD-like lesions. However, its effects on AD-related pathological events have not been investigated. Here, we explored the effects of 1-iodohexadecane on AD lesion-related in vitro and in vivo responses and the mechanism involved using human keratinocytes (HaCaT cells), mast cells (RBL-2H3 cells), and a 2,4-dinitrochlorobenzene (DNCB)-induced mouse model (male BALB/c) of AD. Protein analyses were performed by immunoblotting or immunohistochemistry. In RBL-2H3 cells, 1-iodohexadecane inhibited immunoglobulin E-induced releases of histamine and β-hexosaminidase and the expression of VAMP8 protein (vesicle-associated membrane proteins 8; a soluble N-ethylmaleimide-sensitive factor attachment protein receptor [SNARE] protein). In HaCaT cells, 1-iodohexadecane enhanced filaggrin and loricrin expressions; in DNCB-treated mice, it improved AD-like skin lesions, reduced epidermal thickness, mast cell infiltration, and increased filaggrin and loricrin expressions (skin barrier proteins). In addition, 1-iodohexadecane reduced the β-hexosaminidase level in the serum of DNCB-applied mice. These results suggest that 1-iodohexadecane may ameliorate AD lesion severity by disrupting SNARE protein-linked degranulation and/or by enhancing the expressions of skin barrier-related proteins, and that 1-iodohexadecane has therapeutic potential for the treatment of AD.

## 1. Introduction

Atopic dermatitis (AD) is a common inflammatory disease that is prevalent among all ages and has characteristic symptoms such as severe itching, dry skin, erythema, and scaly, thickened skin lesions [1]. Reportedly, AD is caused by immunological abnormalities and/or epidermal barrier dysfunction, which are associated with the complex interplay between genetic, immunological (increased serum immunoglobulin-E (IgE) levels and imbalance between T helper type 1 (Th1) and Th2 cells) responses, and environmental factors such as environmental pollution [2]. Immunological and skin epidermal barrier functional abnormalities are also closely related to the chronicity and relapse of AD lesions [3]. Therefore, controlling these etiologic factors is a crucial aspect of the development of strategies designed to treat, prevent, or manage AD. Two hypotheses have been proposed to explain the pathogenic mechanism responsible for AD; that is, AD results from epidermal-barrier dysfunction caused by immune system dysregulation, or it is caused by immune dysregulation and inflammation resulting from skin barrier abnormalities [4]. As a result, many researchers seeking to develop new treatments for AD have focused on the skin barrier function and the restoration of the immune balance [5].

Allergic skin diseases such as AD might be associated with mast cells, the major effectors of allergic reactions [6]. These cells release proinflammatory and immunomodulatory mediators, such as histamine and cytokines, through degranulation due to IgE- induced high affinity IgE receptor (FcεRI) activation [7]. Mast cell degranulation is an important component of the allergic response and is caused by the membrane fusion between intracellular vesicles and the outer membranes of the mast cells [8,9]. These membrane fusion events are mediated by SNARE (soluble N-ethylmaleimide-sensitive factor attachment protein receptors) proteins [9,10,11], which are classified as vesicle-associated SNARE (v-SNARE) proteins or target cell-associated SNARE (t-SNARE) proteins based on their cellular localizations in vesicles or target membranes, respectively [10,12]. The t-SNARE protein family includes SNAP (synaptosomal-associated protein) and syntaxin, and v-SNARE proteins include VAMPs (vesicle-related membrane proteins) and synaptobrevin [10,12]. These proteins participate in the regulation of mast cell degranulation via complex formation [12]. Furthermore, the t-SNARE proteins SNAP-23 and syntaxin 4 and the v-SNARE proteins VAMP2, 3, 7, and 8 play key roles in mast cell degranulation [10,11,12]; thus, abnormal complex formation by these proteins can adversely affect mast cell degranulation and possibly epidermal barrier dysfunction [3,13].

The skin barrier protects the body from external challenges by allergens, chemicals, and infectious agents and from excessive transepidermal water loss [14,15]. Skin barrier function is maintained by the stratum corneum of epidermis (the uppermost skin layer). Impaired skin barrier function, the cause of AD, can result in the influx of allergens or irritants and cause skin inflammation by triggering the productions of inflammatory cytokines [14,16] and, thus, exacerbate AD [16,17]. Moreover, it has been reported that aberrant expressions of epidermal differentiation-related molecules such as filaggrin (FLG), loricrin (LOR), and involucrin in AD may disrupt the barrier function of skin [16,18]. The stratum corneum is mainly composed of keratinocytes, which express the two key proteins, FLG and LOR, responsible for epidermal barrier formation and integrity [19,20], and the abnormal expressions of these proteins are related to skin barrier dysfunctions [21,22,23].

Topical and systemic corticosteroids, antihistamines, emollients, and immunosuppressants are currently used to treat AD [3]. Moreover, recently, the use of biologics (e.g., dupilumab) or small molecules (e.g., baricitinib) has been attempted to treat AD [24]. However, the usages of these drugs are limited by their associated side effects [3,24]. In addition, since the pathogenesis of AD is multifactorial, no treatment has been developed that is suitable for all AD patients. Therefore, to address the limitations of current therapies, researchers have focused on the use of plant-sourced agents [25,26,27,28]. 1-Iodohexadecane (also called hexadecyl iodide or cetyl iodide) has been reported in plant extracts with antimicrobial, antioxidant, anticancer, and other bioactivities [29,30,31]. 1-Iodohexadecane (1-Iodo) was identified in the essential oil of the *Chrysanthemum boreale* MAKINO flower, which was suggested to have potential anti-AD activity and to regulate SNARE protein-associated mast cell degranulation and skin barrier-related protein levels [28]. However, the pharmacological and biological activities of 1-Iodo in skin have not been investigated. Thus, we investigated its effects on AD-like lesions in mice exposed to DNCB (2,4-dinitrochlorobenzene), degranulation-associated SNARE proteins in mast cells (RBL-2H3 cells), skin barrier-related proteins in keratinocytes (HaCaT cells), and the responsible mechanism.

## 2. Results

### 2.1. Effects of 1-Iodohexadecane on the Expressions of SNARE Proteins in Mast Cells

**Figure 1:**
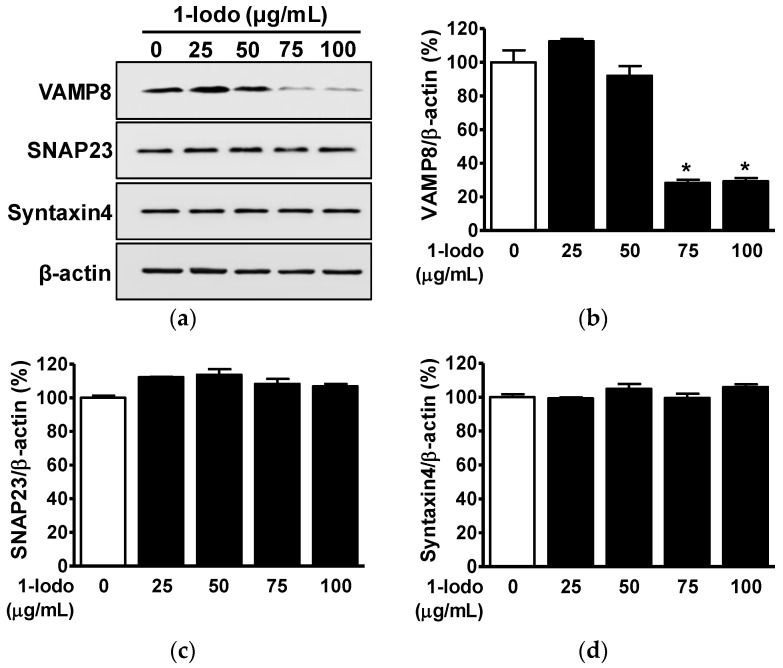
Effects of 1-iodohexadecane on the expressions of SNARE proteins in RBL-2H3 cells. (**a**) RBL-2H3 mast cells were incubated for 48 h in the presence or absence of 1-iodohexadecane (1-Iodo; 25–100 μg/mL). Cell lysates were immunoblotted with the indicated antibodies. (**b**–**d**) Graphs show the expression levels of the VAMP8 (**b**), SNAP23 (**c**), and syntaxin 4 (**d**) proteins shown in panel a. Results are expressed as mean percentages ± SEMs versus untreated cells (*n* = 3). * *p* < 0.05 vs. untreated cells.

SNARE proteins are membrane fusion-regulatory molecules associated with the regulation of mast cell degranulation, which results in the release of inflammatory mediators of AD such as histamine and cytokines [10,11,12,13]. t-SNARE proteins, such as SNAP23 and syntaxin 4, and v-SNARE proteins, such as VAMP7 and VAMP8, control the membrane fusion required for mast cell degranulation [12,32]. To determine whether 1-Iodo affects SNARE proteins, we used Western blotting to examine its effects on the expressions of SNAP23 and syntaxin 4 (its partner protein) and VAMP8 in RBL-2H3 mast cells. Treatment with 1-Iodo (25–100 μg/mL) significantly decreased VAMP8 expression at 75 μg/mL (28.31 ± 1.83% vs. untreated controls) and 100 μg/mL (29.30 ± 1.93% vs. untreated controls) (Figure 1a,b), but 1-Iodo did not influence the expressions of t-SNAREs (SNAP23 and syntaxin 4) in RBL-2H3 mast cells (Figure 1a,c,d).

### 2.2. Effects of 1-Iodohexadecane on Histamine and β-Hexosaminidase Secretion by Mast Cells

**Figure 2:**
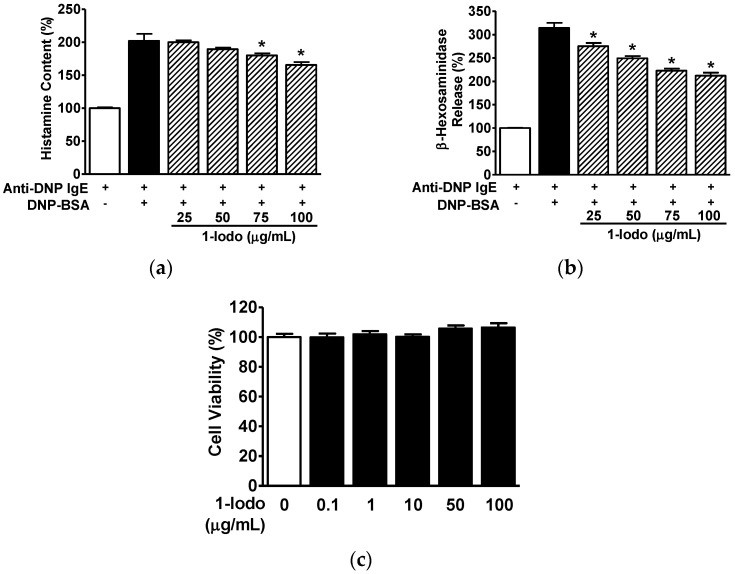
Effects of 1-iodohexadecane on immunoglobulin E-antigen complex-induced histamine and β-hexosaminidase release from RBL-2H3 cells. (**a**,**b**) Histamine and β-hexosaminidase releases from RBL-2H3 mast cells. After treatment for 48 h in the presence or absence of 1-iodohexadecane (1-Iodo; 25–100 μg/mL), cells were incubated with anti-dinitrophenyl-immunoglobulin E (anti-DNP-IgE; 200 ng/mL) for 10 h and then stimulated with 2,4-dinitrophenyl labeled-BSA (DNP-BSA: 10 ng/mL) for 1 h. Culture media were collected and centrifuged, and the levels of histamine (**a**; *n* = 3) and β-hexosaminidase (**b**; *n* = 3) in the supernatant (conditioned media) were measured using an enzyme immunoassay. The response in cells treated with anti-DNP-IgE alone was considered 100%. The results are represented as means ± SEMs. * *p* < 0.05 vs. DNP-BSA stimulated cells in the presence of anti-DNP-IgE alone. (**c**) Cell viability in RBL-2H3 cells exposed to 1-Iodo. Cells were treated with 1-Iodo (0.1–100 µg/mL) for 48 h. Cell viabilities were measured using an EZ-CyTox kit. The response levels are expressed as percentages of levels in untreated cells (*n* = 5). Results are expressed as means ± SEMs. * *p* < 0.05 vs. untreated cells.

Lysosomal β-hexosaminidase and histamine release by mast cells are key markers of mast cell degranulation [33,34]. To examine the effects of 1-Iodo on histamine and β-hexosaminidase release by 2,4-dinitrophenyl labeled-bovine serum albumin (DNP-BSA)-activated mast cells, we assessed histamine and β-hexosaminidase levels in conditioned media of DNP-BSA (10 ng/mL)-stimulated RBL-2H3 cells incubated with an anti-dinitrophenyl-immunoglobulin E (anti-DNP-IgE) (200 ng/mL) antibody. In the presence of DNP-BSA, the histamine levels were elevated (200.72 ± 5.91% vs. anti-DNP-IgE treated controls; Figure 2a), and the β-hexosaminidase levels were elevated (314.25 ± 10.79% vs. anti-DNP-IgE treated controls; Figure 2b). Furthermore, in the concentration range of 25–100 μg/mL, the 1-Iodo concentration dependently reduced histamine and β-hexosaminidase levels in the conditioned media of DNP-BSA-activated cells (Figure 2a,b). Treatment of cells with 100 μg/mL 1-Iodo resulted in maximally reduced levels of histamine (158.46 ± 5.06% vs. anti-DNP-IgE treated controls; Figure 2a) and β-hexosaminidase (212.07 ± 6.52% vs. anti-DNP-IgE alone-treated control; Figure 2b). Notably, treatment with 1-Iodo at concentrations up to 100 μg/mL did not affect the cell viability of RBL-2H3 cells (Figure 2c).

### 2.3. Effects of 1-Iodohexadecane on Skin Barrier Protein Expressions in Human Keratinocytes

**Figure 3:**
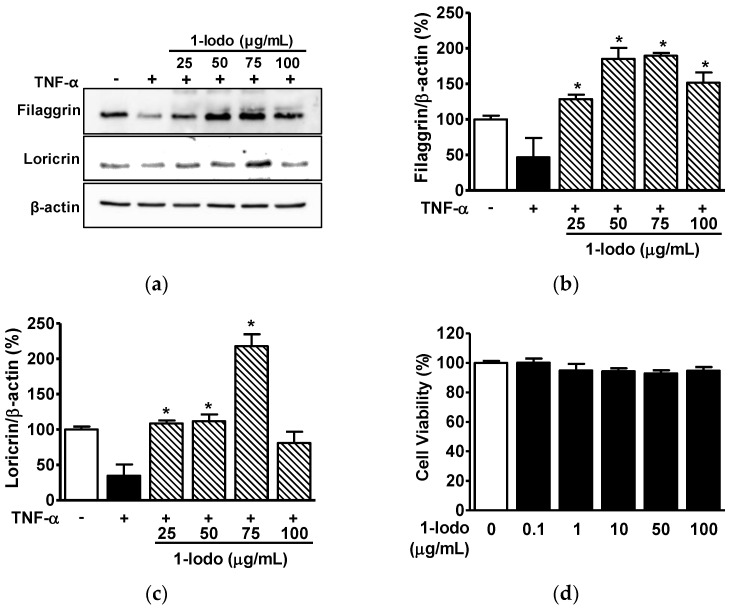
Effects of 1-iodohexadecane on the expressions of skin barrier-related proteins in TNF-α-stimulated keratinocytes. (**a**–**c**) Expressions of skin barrier-related proteins. HaCaT cells were cultured at 37 °C for 36 h in DMEM in the presence or absence of TNF-α (5 ng/mL) with or without 1-iodohexadecane (1-Iodo; 25–100 μg/mL). Cell lysates were immunoblotted with the indicated antibodies. (**a**) Representative images. (**b**,**c**) Results for filaggrin (**b**; *n* = 3) and loricrin (**c**; *n* = 3) were obtained from panel A. (**d**) Cell viabilities. HaCaT cells were treated with 1-Iodo (0.1–100 µg/mL) for 36 h, and viabilities were measured using an EZ-CyTox kit (*n* = 5). Responses are expressed as percentages of untreated cells. The results are represented as means ± SEMs. * *p* < 0.05 vs. TNF-α alone-stimulated cells.

FLG and LOR play important functional roles in the barrier properties of skin [22,35], and low levels of these proteins are associated with skin barrier disruption and AD development [22,35]. Tumor necrosis factor-α (TNF-α) is known to impair the skin barrier by inhibiting FLG and LOR expression in keratinocytes [36]. Thus, to determine the influence of 1-Iodo on FLG and LOR in keratinocytes, we assessed their levels in tumor necrosis factor-α (TNF-α)-stimulated HaCaT cells in the presence or absence of 1-Iodo by immunoblotting. As shown in Figure 3, TNF-α reduced the expressions of FLG (46.51 ± 27.34% vs. untreated controls) and LOR (34.65 ± 16.03% vs. untreated controls). Treatment with 1-Iodo (25–100 μg/mL) significantly prevented TNF-α induced reduction in FLG at concentrations of 25 to 100 μg/mL (Figure 3a,b) and LOR protein levels at concentrations of 25 to 75 μg/mL (Figure 3a,c). These effects were highest at a 1-Iodo concentration of 75 μg/mL (FLG expression, 189.57 ± 3.97% of untreated controls [Figure 3b]; LOR expression, 217.62 ± 16.95% of untreated controls [Figure 3c]). Furthermore, 1-Iodo did not affect HaCaT cell viability at concentrations up to 100 μg/mL (Figure 3d).

### 2.4. Effect of 1-Iodohexadecane on Skin Lesions in the DNCB-Induced Murine Model

**Figure 4:**
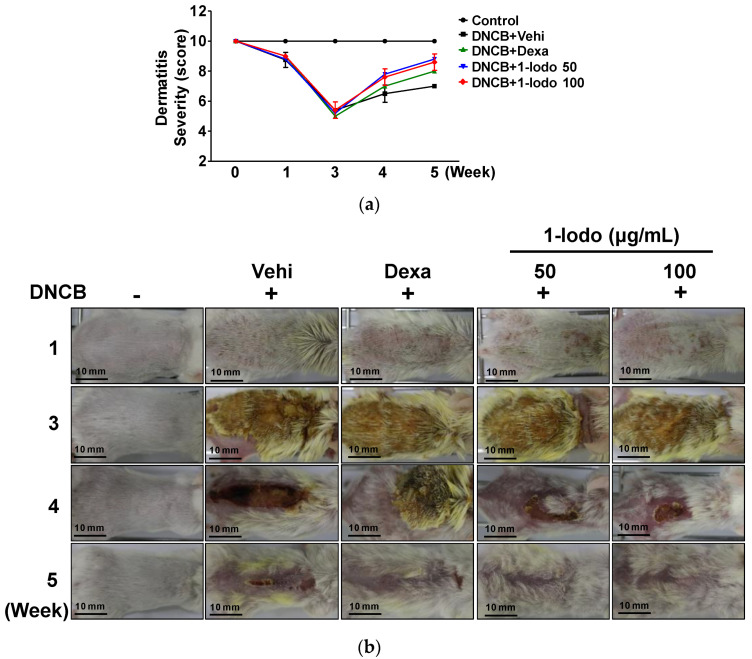
Changes in AD-like lesion severity by 1-iodohexadecane treatment in DNCB-induced mice. The dorsal skins of mice were treated with 2,4-dinitrochlorobenzene (DNCB) with or without 1-iodohexadecane (1-Iodo; 50 or 100 μg/mL) for 21 days. Skin lesions were observed macroscopically and lesion severities were assessed using SCORAD scores as described in the Materials and Methods section. (**a**) Graph of results shown in panel (**b**). (**b**) Representative pictures showing DNCB-induced AD-like skin symptoms before and after treatments. Scale bar = 10 mm. Dexamethasone (Dexa; 0.1%) was used as a positive control (*n* = 5). Control, DNCB-untreated control; Vehi, vehicle (100% olive oil).

To determine the in vivo effects of 1-Iodo, we investigated its effect on the severity of DNCB-induced skin lesions using SCORAD scores. As shown in Figure 4, DNCB reduced SCORAD scores, and these scores were markedly improved in the 50 and 100 μg/mL 1-Iodo groups and in the DEX group.

### 2.5. Histopathological Alterations and Serum Levels of Mast Cell Degranulation Markers in the DNCB-Induced Murine Model Exposed to 1-Iodohexadecane

**Figure 5:**
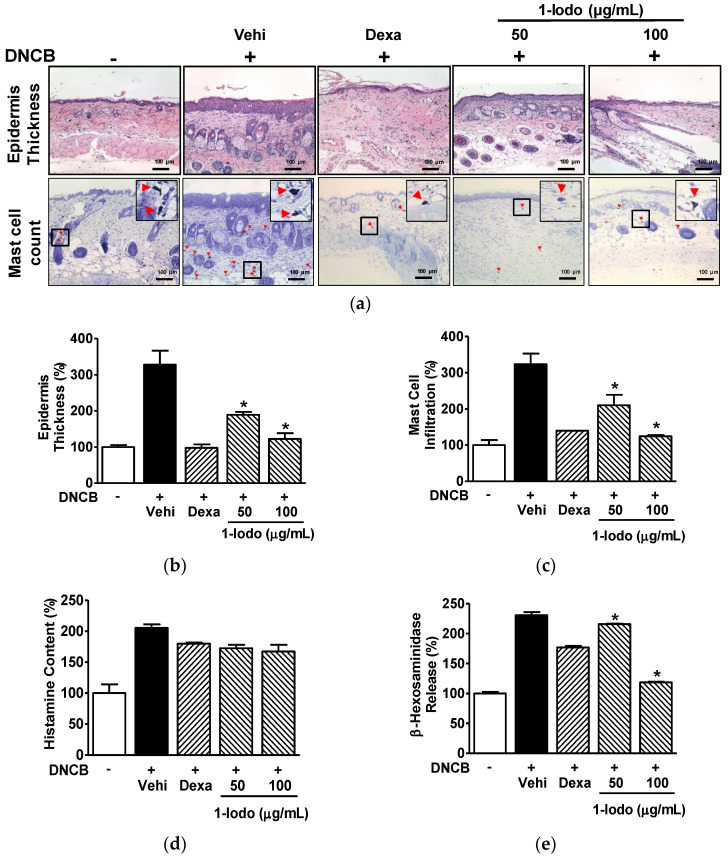
Histopathological features of skin lesions and serum levels of mast cell degranulation markers in DNCB-induced mouse model treated with 1-iodohexadecane. The dorsal skins of mice were treated with 2,4-dinitrochlorobenzene (DNCB) with or without 1-iodohexadecane (1-Iodo; 50 or 100 μg/mL) for 21 days. The skin tissues were excised, fixed with 4% formaldehyde, embedded in paraffin, and sectioned as described in the Materials and Methods section. The blood was collected and centrifuged to obtain the serum as described in the Materials and Methods section. (**a**) Representative histological images. Sections were stained with H&E for epidermal thickness measurements or toluidine blue for mast cell counting. Red arrowheads indicate mast cells stained with toluidine. Scale bar = 100 μm. (**b**,**c**) Graphs of the results shown in panel (**a**). The epidermal thickness (upper images of panel (**a**), *n* = 3) and mast cell infiltration (lower images of panel (**a**), *n* = 3) were measured using Image J software. (**d**,**e**) Serum levels of histamine and β-hexosaminidase. Levels of histamine (**d**; *n* = 2) and β-hexosaminidase (**e**; *n* = 2) in the obtained sera were measured using an enzyme immunoassay. Dexamethasone (Dexa, 0.1%) in each test was used as a positive control. Values are expressed as percentages of those of non-treated controls. The results are presented as means ± SEMs. * *p* < 0.05 versus DNCB controls. Vehi, vehicle (100% olive oil).

Increased epidermal thickness and mast cell infiltration are histological features observed in AD lesions [37,38]. DNCB increased epidermal thickness (327.86 ± 38.60% vs. normal controls), and this increase was significantly attenuated by 1-Iodo at 50 or 100 μg/mL (189.08 ± 7.94% and 122.39 ± 15.76%, respectively, vs. normal controls) (Figure 5a,b). Mast cell infiltration into dorsal skin was greater in the DNCB control group than in normal controls (323.61 ± 29.30% vs. normal controls), and this was reduced to 210.18 ± 28.90% and 124.55 ± 3.31% in the 50 and 100 µg/mL 1-Iodo groups, respectively, vs. normal controls (Figure 5a,c).

In addition, to determine whether 1-Iodo influences mast cell degranulation markers in vivo, we assessed histamine and β-hexosaminidase levels in the serum of DNCB-induced mice model treated with 1-Iodo. DNCB elevated histamine levels (205.36 ± 5.48% vs. normal controls), and this elevation was slightly attenuated by 1-Iodo at 50 or 100 μg/mL (172.47 ± 5.48% and 166.99 ± 10.96%, respectively, vs. normal controls) (Figure 5d). In addition, DNCB increased β-hexosaminidase levels (230.58 ± 5.31% vs. normal controls), and this increase was significantly decreased by 1-Iodo at 50 or 100 μg/mL (215.82 ± 0.93% and 118.48 ± 1.31%, respectively, vs. normal controls) (Figure 5e).

### 2.6. Effect of 1-Iodohexadecane on Skin Barrier Proteins in the DNCB-Induced Murine Model

**Figure 6:**
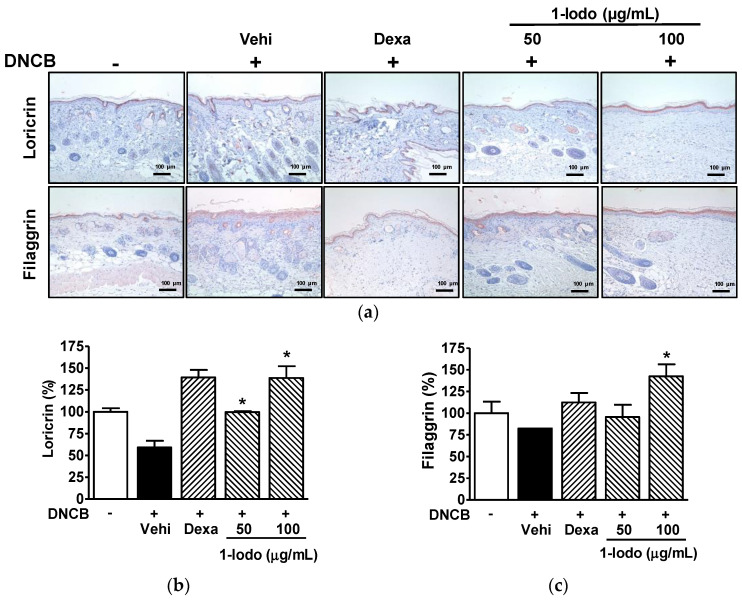
Expressions of skin barrier-related proteins in the skins of mice treated with DNCB and 1-iodohexadecane. (**a**) Representative images. The dorsal skins of mice were treated with 2,4-dinitrochlorobenzene (DNCB) with or without 1-iodohexadecane (1-Iodo; 50 or 100 μg/mL) for 21 days. Skin tissues were excised, fixed with 4% formaldehyde, embedded in paraffin, and sectioned, and sections were then immunostained with anti-loricrin or -filaggrin as described in the Materials and Methods section. Scale bar = 100 μm. (**b**,**c**) Results obtained from panel (**a**). The expression levels of loricrin (upper images of panel (**a**), *n* = 3) and filaggrin (lower images of panel (**a**), *n* = 3) were measure using Image Pro Plus. Protein levels are expressed as percentages of those of DNCB-untreated controls. Dexamethasone (Dexa, 0.1%) was used as a positive control. Values are means ± SEMs. * *p* < 0.05 vs. the DNCB control group. Vehi, vehicle (100% olive oil).

Immunostaining was used to determine whether 1-Iodo affected LOR and FLG levels in lesioned skin tissues. LOR expression in skin was lower in the DNCB control group than in normal controls (59.21 ± 7.53% of normal control levels) but at 99.71 ± 1.02% and 138.70 ± 13.59% of normal control levels in the 50 and 100 µg/mL 1-Iodo groups, respectively (Figure 6a,b). FLG protein levels were lower in the DNCB control group than in normal controls (82.40 ± 0.01% vs. normal controls) but were at 142.50 ± 13.89% of the normal control level in the 100 µg/mL 1-Iodo group. However, 50 μg/mL of 1-Iodo had no effect (Figure 6a,c).

## 3. Discussion

Worldwide, AD has a prevalence of 15–20% among children and of 1–3% among adults, and its prevalence continues to increase [39]. In severe cases, AD can profoundly impair the quality of life and mental health of patients and of relatives, etc. [40]. Available treatments for AD include topical and systemic corticosteroids, antihistamines, emollients, and immunosuppressants [3]. However, these treatments have limitations in overcoming AD. Thus, much research has been conducted to devise ways of treating AD effectively [41]. DNCB-induced animal models have often been used for AD treatment and pathogenesis studies, because they exhibit symptoms with histological and immunological features similar to those of human AD [42]. 1-Iodo is a component of *Chrysanthemum boreale* MAKINO flower essential oil with potential anti-AD activity [28]. Thus, in the present study, we investigated its effects on DNCB-induced AD-like lesions in mice. Similar to the dorsal skin tissues in DNCB-exposed AD-like model mice [27,43], DNCB exposure exacerbated lesion severity, as determined by SCORAD scores, epidermal thickness (hyperplasia), and mast cell infiltration into dorsal skin tissues. These DNCB-induced pathological lesions in mice were improved by topical treatment with 1-Iodo. Therefore, 1-Iodo may have an ameliorative effect on AD-like skin lesions.

Studies have suggested that the regulation of mast cell degranulation by SNARE proteins may be a therapeutic target in AD [13]. Degranulation in mast cells is closely associated with membrane fusion events between vesicles/granules and the plasma membrane and results in the release of allergic inflammatory mediators [9,10]. SNARE proteins, such as v-SNAREs and t-SNAREs, are important players in degranulation-associated membrane fusion in mast cells [9,44]. The t-SNARE proteins SNAP23 and syntaxin 4 are localized in the plasma membrane, and the v-SNARE proteins VAMP7 and VAMP8 are localized on intracellular granules. These two types of SNARE proteins form complexes with each other, and thus, induce membrane fusion between vesicles/granules and the plasma membrane leading to degranulation [9,10,11]. Furthermore, it has been reported that knockdown of SNAP23, syntaxin 4, or VAMP8 reduces mast cell degranulation [9]. In the present study, 1-Iodo reduced the expression of VAMP8 in RBL-2H3 cells but not those of SNAP23 and syntaxin 4. It has been reported that VAMP8 deficiency caused impaired mast cell degranulation and inhibited IgE-stimulated release of β-hexosaminidase and histamine by mast cells [45]. On the other hand, another study showed that VAMP8 deficiency reduced β-hexosaminidase release but not histamine release in mast cells [46]. These two reports indicate VAMP8 may affect mast cell degranulation, though its effects on histamine release effects require clarification. These findings imply 1-Iodo may affect SNARE complex assembly-related degranulation, probably by downregulating VAMP8, and it may suppress the release of degranulation-mediated granule contents by interfering with SNARE complex formation. To the best of our knowledge, these results are the first to indicate 1-Iodo inhibits SNARE protein-associated mast cell degranulation.

Degranulation in mast cells is activated by allergens or IgE and results in the release of inflammatory mediators such as histamine, β-hexosaminidase, and cytokines [7,47]. These inflammatory mediators can induce allergic reaction-linked pathological events, such as skin barrier abnormalities and/or immune dysregulation in AD [10,11,12,13]. These reports indicate that suppressing mast cell degranulation or secretion of histamine and β-hexosaminidase may be a useful strategy to improve the condition of AD. In the present study, 1-Iodo treatment reduced β-hexosaminidase and histamine release, both indicators of mast cell degranulation [32,33], from IgE-exposed RBL-2H3 cells. Moreover, 1-Iodo downregulated VAMP8 expression in RBL-2H3 cells, As mentioned above, VAMP8 play essential role in mast cell degranulation through SNARE complex formation and its defi- ciency impaired mast cell degranulation and inhibited release of β-hexosaminidase and histamine by mast cells [45]. Our findings support the notion that 1-Iodo suppresses the release of degranulation-mediated granule contents by interfering with SNARE complex formation in mast cells. In an animal model of AD, mast cell degranulation inhibition suppressed β-hexosaminidase, histamine, and cytokine secretion and ameliorated AD-like lesions [13,48]. In the present study, we found that topical treatment with 1-Iodo inhibited mast cell infiltrations into dorsal skin tissues in AD-like model mice, implying that mast cells may be associated with skin lesions ameliorated by 1-Iodo in like model mice. Moreover, 1-Iodo significantly reduced the β-hexosaminidase level and slightly attenuated the histamine level in serum of AD-like model mice. Therefore, it can be assumed that 1-Iodo may help alleviate the condition of AD, probably by inhibiting mast cell degranulation-related responses. However, further study will need to clarify the mechanisms involved in the effects of 1-Iodo on these responses in an animal model of AD.

Epidermal skin barrier dysfunction and inflammation play key roles in the development of AD [49], and epidermal structural proteins such as FLG, LOR, and involucrin are key players in epidermal skin barrier formation [50]. FLG is a major cytoskeleton protein of the cornified envelope, and LOR is a main structural cornified envelope protein that constitutes 70–85% of total protein mass in the cornified layer [19]. Involucrin is an assembly and scaffold protein of the cornified envelope [19]. Reduced expressions of these epidermal barrier proteins are major pathological characteristics in the skin of AD patients [50]. Reduced levels of FLG and LOR expressions are associated with barrier disruption and skin inflammation, weakening of the epidermal barrier function, and are also observed in inflamed skin [51,52]. In the present study, treatment with 1-Iodo prevented TNF-α-induced reduction in the expressions of FLG and LOR in keratinocytes and DNCB-induced reduction in the expressions of FLG and LOR in dorsal skins in our murine model. It has been reported that FLG and LOR expressions are decreased by histamine in keratinocytes and human AD skin and suggested that this may be associated with impaired skin barrier function [53]. As mentioned above, we also observed that 1-Iodo inhibited histamine release by IgE-activated RBL-2H3 mast cells Moreover, 1-Iodo decreased mast cell infiltrations into dorsal skin tissues and reduced, but not significantly, serum level of histamine in AD-like model mice. These results suggest 1-Iodo upregulates FLG and LOR by suppressing histamine release by mast cells, and that 1-Iodo might have a positive effect on the skin barrier function.

## 4. Materials and Methods

### 4.1. Materials

Dulbecco’s modified Eagle’s medium (DMEM), fetal bovine serum (FBS), phosphate-buffered saline (PBS), trypsin-ethylenediamine tetra acetic acid (EDTA), and streptomycin/penicillin were purchased from Welgene (Daegu, Korea) and Gibco (Grand Island, NY, USA). Anti-DNP-IgE, bovine serum albumin (BSA), *p*-nitrophenyl-*N*-acetyl-β-d-glucosaminide, DNCB, and 1-Iodo (1-iodohexadecane) were purchased from Sigma-Aldrich (St. Louis, MO, USA). DNP-BSA was purchased from Thermo Scientific (Waltham, MA, USA) and TNF-α from R&D Systems (Minneapolis, MN, USA). Anti-SNAP23, anti-syntaxin 4, anti-VAMP8, anti-LOR, and anti-FLG antibodies were from Abcam (Cambridge, MA, USA), and the anti-β-actin antibodies were from Sigma-Aldrich. Anti-rabbit IgG HRP-conjugated and anti-mouse IgG HRP-conjugated antibodies were from Cell Signaling (Danvers, MA, USA).

### 4.2. Cell Culture

Rat basophilic leukemia (RBL-2H3) cells were purchased from the Korean Cell Line Bank and human skin keratinocytes (HaCaT cells) from the Daegu Gyeongbuk Institute for Oriental Medicine Industry (Daegu, South Korea). Cells were cultured in DMEM supplemented with 10% FBS and antibiotics (100 U/mL penicillin/100 μg/mL streptomycin, and 200 mM glutamine) at 37 °C in a humidified 5% CO_2_ atmosphere.

### 4.3. Cell Viability

Cell viabilities were determined using the EZ-CyTox kit (DAEIL LAB, Seoul, South Korea). RBL-2H3 (5 × 10^3^/well) or HaCaT cells (5 × 10^3^/well) were seeded into 96-well cell culture plates and treated with different concentrations of 1-Iodo for 48 or 36 h, respectively, in a humidified 5% CO_2_ atmosphere and then incubated with EZ-CyTox reagent (10 μL/well) for 30 min in a 5% CO_2_ atmosphere at 37 °C. Cell viabilities levels were determined using a multi-well plate reader (Synergy 2, Bio-Tek Instruments, Winooski, VT, USA) at 450 nm.

### 4.4. Immunoblotting

RBL-2H3 cells (1 × 10^5^ cells/well) or HaCaT cells (1 × 10^6^ cells/well) were incubated in 60 mm or 100 mm culture dishes, respectively, at 37 °C overnight. After washing with PBS, RBL-2H3 cells were treated with test samples for 48 h, whereas HaCaT cells were pretreated with TNF-α and then incubated with test samples for 36 h. After treatments, the cells were washed with PBS and lysed with RIPA buffer (Cell Signaling). Lysates were centrifuged (17,000× *g*, 15 min, 4 °C), and the supernatants were collected. Cellular proteins (30–100 μg/lane) were separated by 8–12% SDS-PAGE and then transferred electrophoretically to polyvinylidene fluoride membranes (Durapore^®^, hydrophilic, 0.45 μm; Millipore, Billerica, MA, USA). Nonspecific binding sites on membranes were blocked for 2 h at room temperature (RT) with PBS containing 3% nonfat dry milk. Membranes were then incubated with the primary antibodies (diluted 1:1000–5000) overnight at 4 °C and reacted with the HRP-conjugated secondary antibodies (diluted 1:1000–5000) at RT for 1 h. Immunoreactive bands were visualized using a chemiluminescent substrate and detected using a chemiluminescent imaging system (LuminoGraph, ATTO, Tokyo, Japan).

### 4.5. β-Hexosaminidase and Histamine Release Assays

In vitro analysis: RBL-2H3 cells (2 × 10^4^ cells/well) were seeded in a 24-well plate and cultured for 12 h. After washing with PBS, cells were incubated with different concentrations of the test sample (1-Iodo) for 48 h and then sensitized with anti-DNP-IgE (200 ng/mL) for 10 h at 37 °C. After washing with Siraganian buffer (119 mM NaCl, 5 mM KCl, 5.6 mM Glucose, 25 mM PIPES, 0.4 mM MgCl_2_, CaCl_2_ 1 mM, 0.1% BSA, pH 7.2), cells were incubated with DNP-BSA (10 ng/mL) for 1 h at 37 °C. Media were collected and centrifuged at 10,000× *g* for 10 min at 4 °C. For β-hexosaminidase release analysis, the supernatants (conditioned media; 50 μL/well) were transferred to a 96-well plate, incubated with 100 μL of the substrate (1 mM *p*-nitrophenyl-N-acetyl-β-D-glucosaminide in 0.05 M citrate buffer, pH 4.5) for 1 h at 37 °C; then, reaction stop solution (0.05 M sodium carbonate buffer (pH 10.0)) was added. Absorbances were measured at 405 nm using an ELISA reader (Synergy 2, Bio-Tek Instruments). For histamine release analysis, supernatants (100 μL/well) were transferred to a 96-well plate and histamine contents were determined using a histamine enzyme assay kit (Cayman Chemical, Ann Arbor, MI, USA). In vivo analysis: Serum was diluted 1:5 in PBS, and then β-hexosaminidase and histamine levels were analyzed as described above for β-hexosaminidase and histamine release analysis in cells. The levels of histamine content and β-hexosaminidase release were expressed as a percentage of the control (anti-DNP-IgE alone group, in vitro; non-treated group, in vivo).

### 4.6. DNCB-Induced Atopic Dermatitis Animal Model

All tests involving animals were conducted in accordance with the Guide for the Care and Use of Laboratory Animals published by the U.S. National Institutes of Health (NIH publication No. 85–23, revised 1996) and approved by the Animal Subjects Committee of Konkuk University, Korea (Approval number: KU20083). The atopic dermatitis was induced in shaved dorsal skin using DNCB as previously reported [27,43].

BALB/c mice (male, 5–6 weeks old; Orient Bio, Korea) were divided into five groups: the normal control group (*n* = 5), a DNCB plus topical olive oil group (*n* = 5; the DNCB control group), a DNCB plus topical 0.1% dexamethasone (*n* = 5; the DEX group), a DNCB plus topical 1-Iodo 50 μg/mL (*n* = 5; the 50 μg/mL 1-Iodo group), and a DNCB plus topical 1-Iodo 100 μg/mL (*n* = 5; the 100 μg/mL 1-Iodo group). DNCB-induced atopic dermatitis in animals was induced using the following protocol. The dorsal skin was shaved 1 day before DNCB sensitization (day 0). For all groups except the normal control group, on days 1, 3, 5, and 7, 200 μL of DNCB (1%; dissolved in acetone and olive oil mixture (4:1, *v*/*v*) was applied to the dorsal skin (the sensitization period). The 1% DNCB-applied dorsal skins were treated with 0.5% DNCB solution daily from day 8 to day 14, to induce dermatitis (the induction period). The dorsal skins of dermatitis-induced mice were treated with 0.2% DNCB plus 100% olive oil (extra pure grade, Duksan, Korea) (the DNCB control group) and with 0.2% DNCB plus 50 μg/mL or 100 μg/mL of 1-Iodo dissolved in 100% olive oil (the 50 and 100 μg/mL 1-Iodo groups) daily from day 15 to day 35. Animals in the DEX group, a positive control group, were also treated with 0.2% DNCB plus 0.1% dexamethasone dissolved in 100% olive oil from day 15 to day 35 in the same manner as treatments were applied in the 50 and 100 μg/mL 1-Iodo groups. In addition, animals in the normal control groups were treated with 100% olive oil daily from day 15 to day 35. On day 35, images of animals were obtained, some animals were sacrificed by CO_2_ inhalation, and skin samples were excised. Other animals were anesthetized with isoflurane, blood was collected by a cardiac puncture, and the animals were sacrificed by CO_2_ inhalation, followed by excision of skin samples. The collected blood was centrifuged for 10 min to obtain the serum. The serum was stored at −80 °C for experiments.

### 4.7. Evaluation of Dermatitis Severity

Dermatitis severity was assessed weekly using SCORAD scores [27,43]. In brief, dermatitis severity was assessed by scoring the severities of edema, erythema/hemorrhage, erosion/excoriation, and dryness, which were awarded scores of 0 to 3 (where a score of 0 indicated severe and a score of 3 indicated no symptom). Dermatitis severity was defined as the sum of these four symptom scores.

### 4.8. Histopathological Analysis

Dorsal skin tissues (3 × 4 cm sizes) prepared from five mice per group were fixed in 4% paraformaldehyde, sectioned at 4 μm, deparaffinized, and stained with hematoxylin and eosin (H&E) to evaluate epidermal hyperplasia (thickness) or stained with toluidine blue to evaluate mast cell infiltration. For immunohistochemistry, staining experiments were conducted using the MicroProbe Manual Staining System (Fisher Scientific, Pittsburgh, PA, USA), some deparaffinized skin sections were immersed in 0.3% H_2_O_2_ to abolish endogenous peroxidase activity and incubated with polyclonal mouse anti-FLG (diluted 1:500) and anti-LOR (diluted 3 μg/mL) for 30 min at 38 °C. Skin sections were then treated with a secondary antibody and 3-amino-9-ethyl-carbacole as required by the UltraVision LP Detection System (Thermo Scientific, Waltham, MA, USA) and counterstained with hematoxylin for 30 s. All sections were photographed under a microscope (BX50F; Olympus Optical Co., Ltd.), and the images were analyzed using Image Pro Plus Version 4.5 software (Media Cybernetics Co., Silver Spring, MD, USA).

### 4.9. Statistical Analysis

Data are expressed as the means ± standard errors of the means (SEMs) of the indicated numbers of experiments. The significances of differences between pairs of groups were determined using the Student’s *t*-test, and multiple comparisons were performed by one-way analysis of variance (ANOVA) followed by the Newman–Keuls post hoc test. The analysis was performed using GraphPad Prism (Version 6.0; GraphPad Software, San Diego, CA, USA), and *p* values of <0.05 were considered significant.

## 5. Conclusions

The present study showed 1-Iodo reduced histamine and β-hexosaminidase release and SNARE protein VAMP8 expression in RBL-2H3 mast cells, and it upregulated FLG and LOR expressions reduced in TNF-α-exposed HaCaT keratinocytes. In vivo, 1-Iodo reduced the severities of dorsal skin lesions, skin epidermal thicknesses, and mast cell infiltration in DNCB lesioned skin, attenuated the DNCB-induced level of β-hexosaminidase in serum, and prevented DNCB-induced reduction in the expressions of FLG and LOR. These findings suggest that 1-Iodo interferes with mast cell degranulation linked to VAMP8 protein and upregulates the expressions of the skin barrier-related proteins FLG and LOR, thus ameliorating AD severity. Therefore, it appears that 1-iodohexadecane is a promising functional material for regulating mast cell degranulation and skin barrier abnormalities. Furthermore, our findings indicate it has therapeutic potential for the treatment of AD. Additional studies are needed to clarify the mechanism responsible for the ameliorative effects of 1-iodohexadecane on AD-like symptoms.

## Data Availability

All the relevant data have been provided in the manuscript.

## Sample Availability

Samples of the compounds are not available from the authors.
